# Medical management of Cushing’s disease: what is the future?

**DOI:** 10.1007/s11102-012-0397-5

**Published:** 2012-06-07

**Authors:** Maria Fleseriu, Stephan Petersenn

**Affiliations:** 1 Departments of Medicine and Neurological Surgery, Northwest Pituitary Center, Oregon Health & Science University, Portland, OR USA; 2 ENDOC Center for Endocrine Tumors, Altonaer Str. 59, 20357 Hamburg, Germany

**Keywords:** Cushing’s disease, Cushing’s syndrome, Pasireotide, Mifepristone, Somatostatin analog, Pituitary adenoma

## Abstract

Cushing’s disease (CD) is caused by a corticotroph, adrenocorticotropic-hormone (ACTH)—secreting pituitary adenoma resulting in significant morbidity and mortality. Transsphenoidal surgery is the initial treatment of choice in almost all cases. Remission rates for microadenomas are good at 65–90 % (with an experienced neurosurgeon) but remission rates are much lower for macroadenomas. However, even after postoperative remission, recurrence rates are high and can be seen up to decades after an initial diagnosis. Repeat surgery or radiation can be useful in these cases, although both have clear limitations with respect to efficacy and/or side effects. Hence, there is a clear unmet need for an effective medical treatment. Currently, most drugs act by inhibiting steroidogenesis in the adrenal glands. Most is known about the effects of ketoconazole and metyrapone. While effective, access to ketoconazole and metyrapone is limited in many countries, experience with long-term use is limited, and side effects can be significant. Recent studies have suggested a role for a pituitary-directed therapy with new multireceptor ligand somatostatin analogs (e.g., pasireotide, recently approved in Europe for treatment of CD), second-generation dopamine agonists, or a combination of both. Mifepristone (a glucocorticoid receptor antagonist) is another promising drug, recently approved by the FDA for treatment of hyperglycemia associated with Cushing’s syndrome. We review available medical treatments for CD with a focus on the two most recent compounds referenced above. Our aim is to expand awareness of current research, and the possibilities afforded by available medical treatments for this mesmerizing, but often frightful disease.

## Introduction

Cushing’s disease (CD) is considered an aggressive pituitary endocrine disorder because of the devastating long-term consequences of untreated hypercortisolemia. Transsphenoidal surgery is clearly the first line therapy of choice; with remission rates of between 65 and 90 % for microadenomas, but somewhat lower rates <65 % for macroadenomas [1]. This variability relates to the size and location of tumors, neurosurgical expertise, length of follow-up, and the definition of remission. Indeed, this last point is a controversial aspect in the management of CD, as there is no consensus on the most accurate postoperative assessment of remission and its predictive value for long-term prognosis [2]. Furthermore, despite initial cure after surgery, up to a quarter of patients will experience recurrence within 10 years [3]. Due to the significance of morbidity and mortality if the disease is not cured [4], patients require additional effective treatment if initial surgery does fail. A second surgery can be a desirable choice only in selected cases [5, 6]. Radiotherapy can also represent an alternative in patients with large remnant or recurrent disease, but can take several months to years to reach biochemical normalization and has inherent adverse effects beyond the risks of panhypopituitarism [7].

Therefore, there is an urgent need for a medical therapy that can effectively reverse clinical features of CD by normalizing CD-associated biochemical parameters acutely—and more importantly during long-term treatment. Although minimally invasive laparoscopic bilateral adrenalectomy is an effective and rapid-onset alternative performed with few short-term complications [8], corticotroph adenoma progression is seen in about 30 % of cases, and may eventually develop to Nelson’s syndrome [9]. Life-long dependence on replacement glucocorticoids and mineralocorticoids may itself be associated with significant morbidity due to recurrent adrenal crises.

**Fig. 1:**
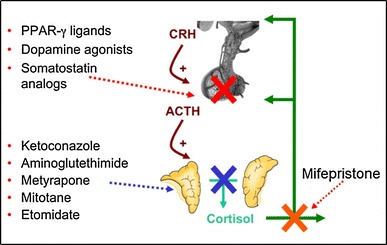
Current approaches to medical therapy in CD, with compounds listed aside their main level of action. Ref. [10] (adapted from Petersenn S, Endocrine Updates, 2011 with kind permission from Springer Science+Business Media B.V.)

Until recently, there have been no available medically licensed treatments, though several compounds had demonstrated efficacy in lowering excess cortisol in selected CD cases [7]. The most physiological approach targets ACTH secretion at the level of the pituitary, with possible inhibition of corticotrope proliferation (Fig. 1, [10]). Pasireotide, a new multiligand somatostatin analog has recently been studied in this regard. Another recent treatment target has been the glucocorticoid receptor. Drugs frequently used now in clinical practice are steroidogenesis inhibitors, which reduce cortisol levels in the adrenal glands. More recently, combination therapy has been also tried with drugs from similar or different groups [11, 12].

Interpretation of available data on the efficacy and safety of most drugs currently used in the treatment of CD is difficult. A review of published reports indicates that study design has varied considerably with a few small prospective, controlled, randomized studies available. Furthermore, it is unclear whether some of those studies could potentially represent publication bias. There is also significant variation in biochemical parameters used for the primary endpoint (e.g., urine free cortisol [UFC], serum and salivary cortisol, and plasma ACTH) and, with few exceptions [13, 14], reference values derived from a sufficiently large population are largely lacking, especially for some of the more recently developed assays. Unfortunately, the criteria for defining a clear and effective response to treatment, and for disease control, are insufficient [1].

## Pituitary-targeted therapy

The aim of pituitary-targeted therapy is to directly act on the underlying pituitary tumor/ACTH hypersecretion source. Although, for many years, various drugs have been tried with limited efficacy, recent approaches may eventually allow for medical treatment of selected patients with CD. Currently, three classes of drugs are under investigation in humans: (1) somatostatin analogs, (2) dopamine agonists, and (3) PPARγ ligands.

### Somatostatin analogs

Table 1 [10] summarizes several studies investigating the expression of the 5 somatostatin receptor subtypes in corticotropic pituitary adenomas [15–21]. Although *sst1*, *sst2*, and *sst5* are widely expressed, the expression levels are low, except for *sst5* [18]. Interestingly, however, both *sst2* ligands and *sst5* ligands were found to inhibit corticotropin-releasing hormone (CRH)-stimulated ACTH secretion in vitro in a mouse corticotropic cell model [22]. Stalla et al. demonstrated clear efficacy of the *sst2* ligand octreotide in primary cell culture of corticotropic pituitary tumors [23]. This effect, however, was abolished by pretreatment with glucocorticoids, which may be explained by downregulation of *sst2* by glucocorticoids. A study of a transient transfection system suggested a negative glucocorticoid responsive element in the *sst2* promoter [24], indicating transcriptional inhibition of *sst2* by glucocorticoids. Assuming inhibition of *sst2* expression in the corticotropic pituitary tumor by continuously elevated systemic cortisol levels, *sst2* ligands would be largely ineffective in vivo. Indeed, although the clinical experience is limited, single injections of octreotide 100 μg did not demonstrate any effect on ACTH levels in several studies of patients with hypercortisolism [23, 25, 26]. Furthermore, short-term treatment of patients with CD with repeated subcutaneous injections of octreotide proved to be largely ineffective [27, 28].

**Table 1:** Expression of somatostatin receptors in corticotropic pituitary adenomas

Reference	Method	*sst1*	*sst2*	*sst3*	*sst4*	*sst5*
Greenman [16, 17]	RPA, RT-PCR	1/3	0/3	1/2	0/1	1/1
Miller [19]	RT-PCR	3/5	5/5	0/5	0/5	4/5
Nielsen [20]	RT-PCR	0/1	0/1	1/1	1/1	0/1
Panetta [21]	RT-PCR	1/1	1/1	1/1	0/1	0/1
Batista [15]	Realtime RT-PCR	12/13 (high)	9/13 (low)	0/13 (-)	5/13 (?)	13/13 (high)
Hofland [18]	Realtime RT-PCR	1/6 (low)	6/6 (low)	2/6 (low)	2/6 (low)	6/6 (high)
Author’s data (SP)	Realtime RT-PCR	7/10 (low–high)	8/10 (low–high)	3/10 (med)	4/10 (high)	7/10 (low–high)
Total (%)		25/39 (64 %)	29/39 (74 %)	8/38 (21 %)	12/37 (32 %)	31/37 (84 %)

Ref. [10] (adapted from Petersenn S, Endocrine Updates, 2011 with kind permission from Springer Science+Business Media B.V.)

**Fig. 2:**
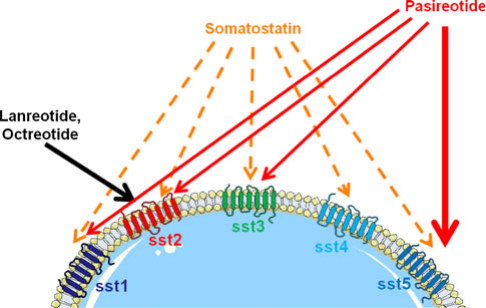
Preferential affinities of somatostatin, somatostatin analogs octreotide and lanreotide, and the new multi-receptor ligand somatostatin analog pasireotide for the five known somatostatin receptor subtypes *sst1*–*sst5*. Ref. [10] (adapted from Petersenn S, Endocrine Updates, 2011 with kind permission from Springer Science+Business Media B.V.)

An alternative approach may be to use *sst5* ligands, considering the high expression of *sst5* in corticotropic adenomas. Pasireotide (SOM230) is a recently developed multi-receptor ligand somatostatin analog. Whereas octreotide and lanreotide have high affinity for *sst*_*2*_ and modest affinity for *sst*_*5*_, pasireotide demonstrates high binding affinity for *sst*_*1,2,3*_ and *sst*_*5*_, and has a 40-fold higher affinity for *sst*_*5*_ than octreotide (Fig. 2, [10, 29, 30]). Pasireotide was highly effective in lowering ACTH secretion in a mouse cell model. Of note, dexamethasone pre-treatment did not influence the sensitivity of the cells to the inhibitory effect of pasireotide, suggesting that *sst5* is relatively resistant to negative control by glucocorticoids [18]. Indeed, quantitative PCR analysis showed that *sst5* mRNA levels were not significantly affected by dexamethasone treatment, whereas dexamethasone lowered *sst2* mRNA expression significantly [31]. In primary cultures of corticotropic pituitary adenomas, pasireotide inhibited ACTH secretion in 3/5 [18] and 5/6 [15] tumors, respectively. In addition, significant suppression of cell proliferation was observed in all tumors cultured in the later study. The strong inhibition of the hypothalamic–pituitary–adrenal (HPA) axis by pasireotide was confirmed in an animal model. Pasireotide suppressed both CRH-induced ACTH release and corticosterone secretion in rats [32]. By overexpression of either *sst2* or *sst5* in a mouse cell model, it was clearly shown that the suppressive effects of pasireotide in corticotropic cells are determined by *sst5*, whereas the ligand action on *sst2* is negligible [33]. In a phase II, proof-of-concept, open-label, single-arm, multicenter study, the in vivo efficacy of pasireotide was evaluated in patients with either de novo CD, or with persistent or recurrent CD [34]. A total of 39 patients were recruited from ten centers in five countries. Approximately 44 and 21 % of patients had a history of a micro- or macroadenoma, respectively, with no visible adenoma or unknown adenoma status in the remaining patients. Baseline UFC levels ranged from 291 to 5,950 nmol/24 h, with a mean of 1,231 nmol/24 h—approximately 4.5 times the upper limit of normal. Thirty-eight patients completed the study, while one patient with a preexisting history of diabetes mellitus discontinued treatment because of grade 2 hyperglycemia. Data from 29 patients were available for the primary efficacy analysis. Five patients had fewer than two UFC sample determinations at baseline or study end, and four patients fulfilled inclusion criteria but had a baseline mean UFC level within the normal range of the central laboratory assay in contrast to increased UFC levels in the local assay. Patients self-administered pasireotide 600 μg subcutaneously twice daily for 15 days, at 0900 and 2100 hours. After the treatment period of 15 days, the mean UFC level decreased significantly by 44.5 %. The mean UFC level significantly decreased from 1,231 nmol/24 h at baseline, to 683 nmol/24 h at study end. Normalization of UFC was found in 17 % (5/29) of patients, with 76 % (22/29) of patients demonstrating a reduction in UFC levels. Although a significant correlation between baseline UFC level and UFC level at day 15 was not observed, there was a trend towards a lower baseline UFC level reported as being predictive of a response to pasireotide. Pharmacokinetic data for pasireotide showed an approximately 1.8-fold higher trough plasma concentration and a 1.3-fold higher plasma exposure in UFC responders than nonresponders. Therefore, the clinical response may be related to pasireotide plasma exposure, requiring pasireotide trough level or plasma exposure above a certain threshold for an optimal clinical response. Steady-state levels of pasireotide were achieved within five days of treatment initiation. Reported side effects with a frequency of at least 10 % included GI-related conditions (diarrhea [44 %], nausea [23 %], abdominal pain [18 %]), asthenia (13 %) and fatigue (10 %), hyperglycemia (36 %), headache (18 %), as well as hypotension (13 %). These very much resemble the side effects observed with currently licensed somatostatin analogs. Hyperglycemia was reported in 36 % (14/39) of patients, and appeared to be more notable in patients with a history of impaired fasting blood glucose or diabetes mellitus.

**Fig. 3:**
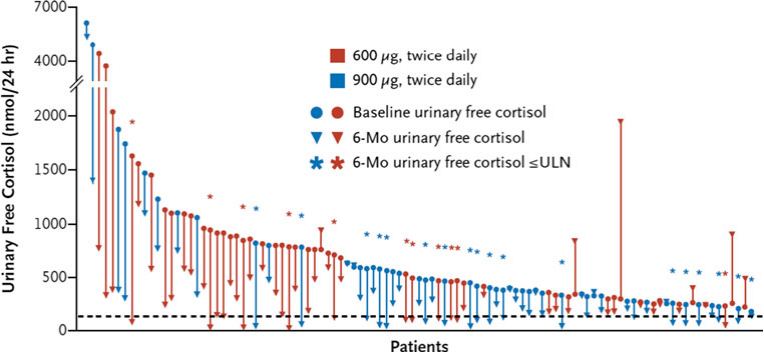
Absolute change of UFC from baseline to month 6 in 103 patients treated with pasireotide. *Black dashed line* represents ULN. Ref. [34]. Reproduced with kind permission from the Massachusetts Medical Society

**Fig. 4:**
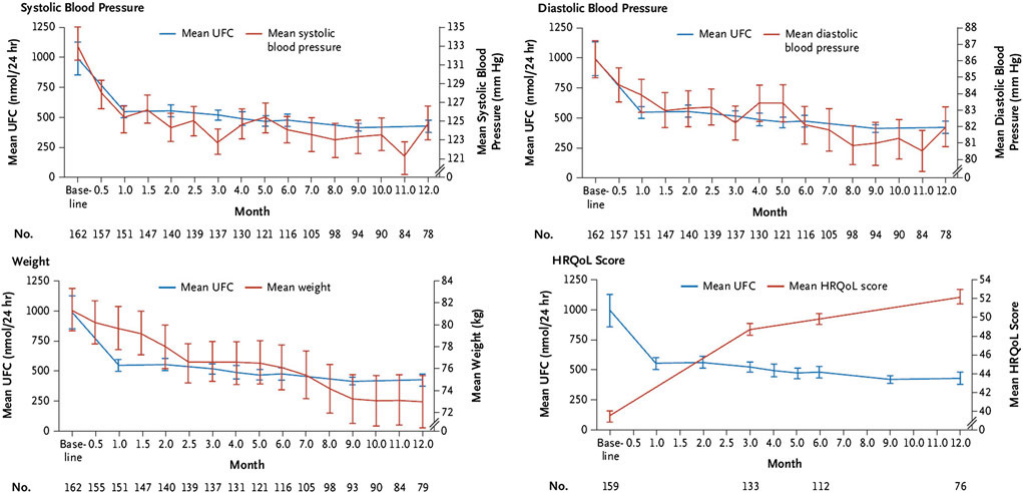
Change from baseline to month 12 in systolic blood pressure, diastolic blood pressure, body weight, and health-related quality of life (HRQoL) (measured using CushingQoL questionnaire, scored from 0 to 100, with higher scores indicated better QoL) among patients treated with pasireotide. *Bars* indicate SEM. Ref. [34] (adapted from Colao A, et al. N Engl J Med 2012). Reproduced with kind permission from the Massachusetts Medical Society

More recently, the results of a phase III, randomized, double-blind, multicenter trial on pasireotide in CD were published [35]. In that study, 162 patients with persistent or recurrent, or de novo CD ineligible for surgery were randomized to receive pasireotide 600 μg or 900 μg subcutaneously bid for 12 months. Hypercortisolism had to be confirmed with a mean baseline UFC (based on four collections) ≥1.5× the upper limit of normal. Key exclusion criteria included pituitary irradiation within the last 10 years, compression of the optic chiasm, and poorly controlled diabetes mellitus. At three months, patients with a mean UFC >2× the upper limit of normal or higher than baseline were unblinded and the dose increased by 300 μg. The primary endpoint was the proportion of patients at six months achieving normalization of UFC without dose up-titration at month 3 relative to the randomized dose. The authors reported that 15 and 26 % for the 600 and 900 μg doses, respectively, met the primary endpoint (Fig. 3, [35]). The lower normalization rate in the 600 μg bid dose group was considered to be due to the higher baseline mean UFC in that group (1,156 vs. 782 nmol/24 h in the 900 μg bid group). Both groups included mainly patients with severe disease. There was a clear reduction in median UFC at 12 months of −67.6 % (600 μg) and −62.4 % (900 μg). Interestingly, lack of response could be identified within 2 months in the vast majority of patients. Serum and salivary cortisol, and plasma ACTH decreased in both groups. As UFC levels decreased, clinical improvements (e.g., with respect to blood pressure, body weight, health-related quality of life) were evident at month 6 and month 12 (Fig. 4, [35]). The most frequently reported adverse events were diarrhea (58 %), nausea (52 %), hyperglycemia (40 %), and cholelithiasis (30 %); most were Grade 1/2. Fasting plasma glucose and HbA_1C_ levels were increased during treatment with pasireotide, and 73 % of patients had a hyperglycemia-related adverse event. The mean HbA_1C_ increased from 5.8 % in both the 600 and 900 μg groups at baseline to 7.2 and 7.4 %, respectively, at month 6. Therefore, patients require careful monitoring of their glucose levels. Following on these results, the European Medicines Agency approved pasireotide in April 2012 for the treatment of adult patients with CD for whom surgery is not an option or for whom surgery has failed [36].

In a recent case report, a dramatic drop of elevated UFC levels in a patient treated with pasireotide 600 μg bid was observed, leading to symptomatic and biochemical hypocortisolism [37]. Prior to pasireotide, the patient demonstrated UFC levels approximately 1.5-2-fold above normal, despite three surgical attempts and radiotherapy. After 10 months of reduced pasireotide therapy (300 μg bid) the patient continued to demonstrate improved mood, stable weight and normal UFC levels. Bode et al. [38] reported a female patient with a giant adenoma that was initially treated by transcranial resection. Due to a symptomatic relapse and tumor recurrence, the patient received stereotactic radiosurgery, followed by bilateral adrenalectomy. Subsequently, the patient was diagnosed with a pituitary carcinoma due to the development of several intracranial and spinal metastases. She was started on temozolomide, with another dramatic rise in ACTH levels (from 882 to 3,868 ng/mL) early after initiation of therapy. As a salvage therapy, she received pasireotide 900 μg subcutaneously bid. ACTH levels decreased to 1,738 ng/mL after 2 weeks. ACTH levels than remained at 900–1,000 ng/mL, and her clinical status improved. The combination of temozolomide and pasireotide led to sustained tumor control for 12 months, after which temozolomide was stopped. The patient then remained stable on monotherapy with pasireotide for more than nine months. The authors themselves state that they cannot dissect the efficacy of both therapies. However, the combination of temozolomide and pasireotide may be a promising option for the treatment of ACTH-secreting pituitary carcinomas.

### Dopamine agonists

Dopamine subtype 2 receptors are expressed in corticotroph pituitary adenomas [39]. Bromocriptine has been shown to inhibit ACTH secretion [40] and apoptosis in an ACTH-secreting mouse cell line [41]. In a combined review of 23 patients with CD treated with 1.25–30 mg/day of bromocriptine for 3–180 weeks, a response rate of 48 % [42] was observed. Of note, primary endpoints for individual studies have varied (e.g., plasma cortisol levels, cortisol secretion rates, and 24-h urinary glucocorticoid assessments). Subsequent series have demonstrated significantly lower response rates (4–23 %) [43, 44]. Newer dopamine agonists may be more effective. In a single-center study of ten patients treated with cabergoline (1–3 mg/week for 3 months), a normalization rate of 40 % for UFC was observed [45]. Moreover, the same authors confirmed the long-term efficacy of cabergoline in CD in a subsequent study, in which normalized UFC was maintained in 40 % of 20 patients for at least 12 months of follow-up [46]. A dose of 1–7 mg/week (median 3.5 mg) was well tolerated, except by two patients with hypotension and severe asthenia, who stopped treatment after 12 and 18 months, respectively. This data was recently confirmed by Godbout et al. [47], who treated 30 CD patients with cabergoline, up to 6 mg/week. Thirty percent of patients demonstrated long-term normalization of UFC for 12–60 months, with a mean dose of 2.1 mg/week. Whether or not recently documented cardiac valve changes in patients with Parkinson’s disease receiving much high doses of cabergoline [48, 49] equate to the lower doses applied in this cohort is not clear. Of note, Pivonello et al. [46] documented no development of cardiac valve insufficiency and one case of worsening of a previously diagnosed tricuspid regurgitation. The results of this single-center experience need to be confirmed in a larger multi-center study that examines both efficacy and safety.

### PPARγ (peroxisome proliferator-activated receptor-gamma)

PPARγ (peroxisome proliferator-activated receptor-gamma) is a member of the nuclear receptor superfamily, and functions as a transcription factor. PPARγ ligands have been shown to inhibit the growth of many tumors, including breast, colon, and prostate cancer cells [50]. Furthermore, PPARγ ligands have demonstrated antiproliferative and apoptotic effects in a murine cell model of corticotropic adenoma cells, and inhibited proopiomelanocortin (POMC) transcription [51]. However, very little PPARγ protein was found in pituitary adenomas. Additionally, the antiproliferative effects of PPARγ ligands were only observed with very high doses of rosiglitazone, without significant reversal by a PPARγ antagonist [52]. In CD, suppression of steroidogenesis in the adrenal glands via inhibition of P450c17 and 3ßHSD could also be beneficial [53], however clinical experience is limited so far. In a study of rosiglitazone (8–16 mg) in 14 patients treated for 1–7 months, normalization of UFC was observed in 42.9 % of patients, with mild improvement in clinical features [54]. A subsequent study in ten patients treated for 1–8 months with 4–16 mg rosiglitazone demonstrated normalization of UFC in 30 % of patients [55]. Side effects, however, were significant and included edema, hypertension, weight gain, somnolence, increased hirsutism, and bruisability. Thus, patients were unwilling to re-enter the treatment protocol [55]. Morcos et al. [56], in a report on long-term treatment of central Cushing’s syndrome with rosiglitazone observed an initial decrease in ACTH, but with a rebound after 28 weeks of therapy, despite dose increases of up to 28 mg. Suri and Weiss published a report on the effect of pioglitazone on ACTH and cortisol secretion in CD. They showed that pioglitazone at 45 mg for one month had no noteworthy effects on either ACTH or cortisol secretion. This study was, however, undertaken in a small study cohort [57]. At a consensus conference, in the investigator panel’s expert opinion, it was concluded that current study results did not sufficiently supported routine clinical use of PPARγ ligands to treat CD [1]. Additionally, there are concerns related to PPARγ ligands and increased cardiovascular disease risks [58], which means that PPARγ ligands are not the ideal drug of choice for patients with Cushing’s who are already at high risk of heart problems.

## Glucocorticoid receptor antagonist therapy

Use of a glucocorticoid receptor blocker represents another medical therapy approach to treat CD [59]. Hypercortisolemia persists, but the devastating effects are ameliorated by the antagonist binding to the glucocorticoid receptor—a concept that is similar to the use of pegvisomant when treating acromegaly [60]. Mifepristone is currently the only available glucocorticoid receptor antagonist [61–63]. Mifepristone directly blocks the cortisol glucocorticoid receptor (GR-II) and the progesterone receptor (PR). There are 51 published case reports detailing the use of mifepristone in treating hypercortisolism (five in CD patients) [64, 65]. Chu et al. [66] presented a case report on a patient with an ACTH-secreting pituitary macroadenoma who had failed multiple therapies and was too ill to undergo bilateral adrenalectomy. A dramatic improvement in clinical symptoms was reported with mifepristone (starting dose 6 mg/kg/day, up to a maximum of 25 mg/kg/day) over 18 months. Severe hypokalemia (which was responsive to spironolactone administration) was attributed to excessive cortisol activation by the mineralocorticoid receptor. In a retrospective report (from seven European centers) on the use of mifepristone in patients with hypercortisolism, four additional patients with CD were presented [65]. Clinical symptoms improved in three during treatment over 3–24 months. The fourth patient was treated for 0.5 months, during which rapid improvements in psychiatric symptoms were demonstrated. The patient later underwent surgery. Severe hypertension and hypokalemia developed in one patient.

**Fig. 5:**
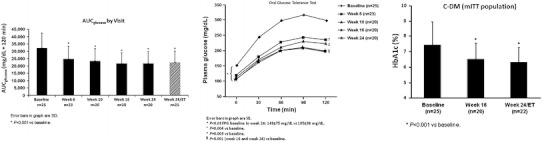
Mean change from baseline to week 24/ET in glycemic parameters (AUC_glucose_ and plasma and fasting plasma glucose as measured by 2-h OGTT, and HbA_1c_) among patients treated with mifepristone. *Bars* indicate SD (AUC, area under the curve; C-DM, Cushing's with diabetes mellitus or hyperglycemia; OGTT, oral glucose tolerance test; ET, early termination) [67] (adapted from Fleseriu M, et al. J Clin Endocrinol Metab 2012. Copyright 2012, The Endocrine Society)

**Fig. 6:**
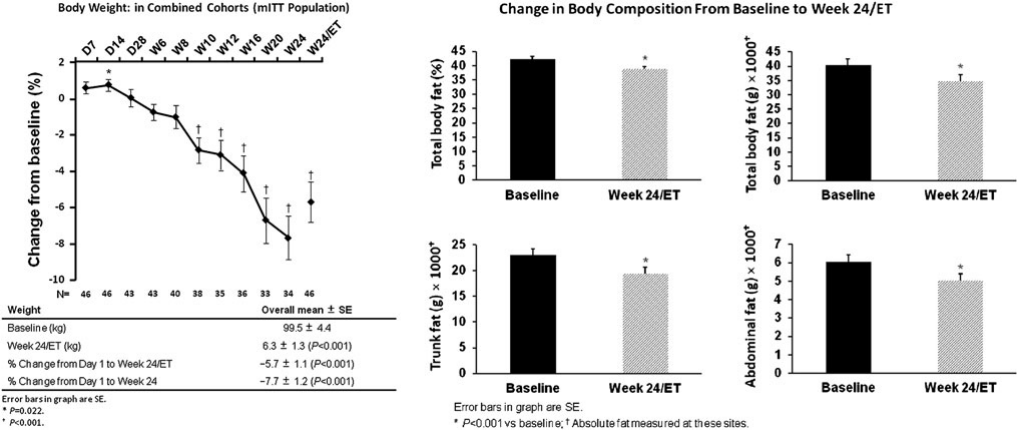
Mean change from baseline to week 24/ET in body weight and composition among patients treated with mifepristone. *Bars* indicate SEM [67] (mITT, modified intent to treat) (adapted from Fleseriu M, et al. J Clin Endocrinol Metab 2012. Copyright 2012, The Endocrine Society)

The multi-center, open-label, six-month United States SEISMIC study included 50 subjects (43 with CD) [67]. Subjects with confirmed endogenous Cushing’s syndrome were placed in two groups, based on the presence of additional eligibility criteria: type 2 diabetes mellitus or impaired glucose tolerance (C-DM cohort), or hypertension (C-HT cohort). Doses of daily mifepristone ranged from 300 to 1,200 mg, with a mean of 900 mg. Overall, 60 % of the 29 patients with glucose intolerance or diabetes (*p* < 0.0001) were defined as responders (a ≥ 25 % reduction in glucose on a standard oral glucose tolerance test as measured from baseline to 24 weeks). There was a continued improvement in glucose tolerance measured at each of the evaluations at weeks 6, 10, 16, and 24 (Fig. 5 [67]). Of the 12 patients taking insulin at baseline, seven were able to cut their daily dose by ≥50 %. There was also a statistically significant reduction in mean HbA_1c_ over the course of the study, from 7.43 % at baseline to 6.29 % at study conclusion (*p* < 0.001). Of the 21 patients enrolled in the hypertension group, 38 % (*p* < 0.05) achieved a 5 mm reduction in diastolic blood pressure. Increases or additions of antihypertensive medications were not permitted with the exception of mineralocorticoid receptor antagonists. Among the 40 patients with hypertension (from both groups), 42.5 % had a reduction in diastolic blood pressure ≥5 mmHg. Fifty-two percent had either a decrease in diastolic blood pressure or a reduction in antihypertensive medications at week 24. Over half of the patients experienced weight loss of ≥5 % when compared to baseline (*p* < 0.001). Mean percent total body fat declined by 3.6 % by week 24 (*p* < 0.001) (Fig. 6 [67]). Eighty-seven percent of patients receiving mifepristone in the study experienced improvement in their individual clinical manifestations (assessed by eight clinical parameters by a blinded data review board). Elevation in cortisol (up to sevenfold) and ACTH (up to twofold) was observed in all patients with CD, and returned to baseline after stopping drug. There were two cases of adrenal insufficiency (AI), although symptoms compatible with AI (e.g., weakness, nausea, fatigue, abdominal pain, emesis, hypotension) were more frequent. Use of glucocorticoid rescue was limited to six patients. Hypokalemia (as expected) was common but generally mild to moderate and associated with alkalosis and edema. There were three cases of severe hypokalemia (defined as K ≤ 2.5 mmol/L) during treatment. All cases responded well to potassium replacement and spironolactone, albeit at significantly high doses. Given that mifepristone has antiprogestin effects, endometrial effects were expected. An increase in endometrial thickness was observed in 38% of the females in the study. There were five cases of vaginal bleeding, two with prolonged bleeding even after stopping drug, and three women underwent subsequent dilatation and curettage (D&C) for non-resolved endometrial thickening after stopping mifepristone. Overall, for most study patients, this drug had an acceptable benefit-risk profile.

The lack of a biochemical marker correlating with the treatment response dictates that efficacy measurements should be based on symptoms, clinical features, and metabolic improvements. Of note, direct translation of clinical study data to day-to-day use in a busy clinical practice can be challenging. Monitoring side effects like hypokalemia and hypertension, as well as early recognition of clinical AI, is essential. Long-term effects of mifepristone on tumor size and endometrial effects are as yet undetermined, and further long-term studies are ongoing [68]. The development of a selective glucocorticoid receptor without antiprogestin effects [69] could also represent an important step in the long-term treatment of women with CD.

## Adrenal-directed therapy: steroidogenesis inhibitors

Steroidogenic drugs have been particularly useful in treating patients with severe hypercortisolism who are waiting for surgery, after noncurative surgery, or as bridge until radiation therapy becomes effective.

The principle of steroidogenesis inhibition originated 60 years ago with studies on amphenone B [70, 71]. To date, most is known about the effects of metyrapone, ketoconazole, and mitotane, which are reportedly more effective and better tolerated, but other drugs have been studied (Table 2). A combination of these drugs may have an additive or synergistic effect, potentially allowing for smaller doses. Treatment monitoring is necessary for hypocortisolemia and any other adverse effects. An approach sometimes used is to totally block adrenal steroidogenesis and add physiologic glucocorticoid replacement [72].

**Table 2:** Adrenal steroidogenesis inhibitors

Drug	Initial dose	Maximal dose in CD
Ketoconazole	200 mg bid	400 mg tid
Metyrapone	250 mg qd	1,500 mg qid
Mitotane	500 mg tid	1,000 mg tid
Etomidate	Bolus of 0.03 μg/kg iv, followed by infusion 0.1 mg/kg/h	0.3 mg/kg/h

Mitotane doses may need to be reduced after 3 months due to saturation of fat tissue and subsequent overspill, look for minimal efficious dose and avoid drug levels >20 μg/mL

### Metyrapone

Metyrapone was studied in humans soon after the discovery of its steroidogenesis blocking effects in animals [73]. It has been used as monotherapy or in combination. Metyrapone mainly acts via inhibition of 11-β-hydroxylase and to a lesser extent 17-α-, 18-, and 19-hydroxylase activities [74] and therefore, aldosterone biosynthesis is more severely affected than that of cortisol [75]. Initial doses are usually 250 mg qid, which can be adjusted during treatment to a total daily dose of 500–6,000 mg [1]. In patients with CD, a significant drop of cortisol levels is observed 2 h after a first dose [76]. Short-term metyrapone therapy induces clinical improvements in the majority of patients, with biochemical control in 75 % (median dose of 2,250 mg/day; range 750–6,000 mg/day). Long-term use (median 27 months, range 3–140 months) induced even higher rates of biochemical remission (83 %), most likely due to an additive effect of pituitary irradiation. Jeffcoate et al. [77], reported biochemical control of 13 patients with CD for up to 66 months with metyrapone (nine had received pituitary irradiation in addition to metyrapone. ACTH secretion may override steroidogenic blockade in some patients with CD [39]. However, as reported in one of the largest studies on metyrapone, this effect predominantly occurred in the first 4–6 weeks following initiation of therapy, after which no further increase was observed [76].

Adrenal insufficiency has been observed in 13 % of patients [76]. Acne and hirsutism (19–70 %) due to increased androgens are relatively frequent, though mostly mild in nature [77], thus making metyrapone a clear second choice in women. As a result of increased 11-deoxycorticosterone levels, hypokalemia (6 %), edema (8 %) and hypertension are also observed [76]. Other rare adverse effects included cutaneous rash (4 %), which may be transient, effects on the central nervous system (e.g., lethargy and dizziness, 15 %), and gastrointestinal complaints (e.g., nausea, 5 %). Metyrapone has limited availability in many countries (e.g., Germany, United States), but can be ordered through an international drugstore or by contacting the manufacturer (Novartis) directly.

### Ketoconazole

Ketoconazole, initially licensed as an antifungal agent, was observed to lower cortisol [78] and testosterone [79] levels via inhibition of a variety of cytochrome P450 enzymes (side-chain cleavage complex, 17,20-lyase, 11-β-hydroxylase, and 17-α-hydroxylase) [80, 81]. Inhibition of ACTH secretion or action as a glucocorticoid receptor antagonist have been described in cell models, but there is a lack of clear evidence in humans [1, 42]. Ketoconazole treatment is usually started at 200 mg bid [1], and biochemical effect achieved at 600–800 mg/24 h [42]. A meta-analysis of 12 studies treating 85 CD patients reported 81 % normalization of urinary steroids [42]. A recent retrospective analysis found normalization of UFC in 45 % of patients (mean follow-up of 23 months; range 6–72 months) [82]. Escape from pharmacological control has been reported [83]. Rapid improvement in clinical symptoms, and regression of diabetes mellitus, hypokalemia, hypertension, hirsutism, and depression have been reported [75]. Ketoconazole may reduce cholesterol [84] and vitamin D levels [85]. Side effects are, in general, dose-dependent and include: gynecomastia (13 % of males), gastrointestinal symptoms (8 %), edema (6 %), and skin rash (2 %) [39]. Ketoconazole represents a second-line in men due to the possible development of hypogonadism. Liver function should be monitored carefully, but mild and transient elevations in liver enzymes of up to threefold of normal are not a contraindication for further treatment. Typically, onset of ketoconazole-induced hepatitis is reported to occur within 60 days after initiation of treatment and resolves within 3 months of discontinuation [86]. More severe abnormalities such as severe hepatic injury (seen in 1/15,000 cases) [87] require discontinuation [1].

The availability of ketoconazole is also limited in many countries. However, fluconazole appears to have similar effects [88], with normalized UFC levels (in the author’s [SP] limited experience with two patients treated with fluconazole 100 mg bid).

### Mitotane (o,p′-DDD)

Mitotane inhibits steroidogenesis at the steps of side chain cleavage, 11- and 18-hydroxylase and 3-β-hydroxysteroid dehydrogenase. Its main use is in treatment of adrenocortical carcinoma, but has been proven effective in CD [89]. In a study of 46 patients treated for 3–34 months at doses of 8–12 g/24 h (different formulation than currently available preparations, which require lower doses), 83 % achieved normalization of urinary markers [90]. Sixty percent of patients relapsed after withdrawal of therapy, suggesting adrenolytic action is limited [90, 91]. Action is slow and saturation can be expected 2–3 months after initiation of therapy. Due to an accelerated metabolism of exogenous steroids, especially hydrocortisone, replacement doses must be increased to avoid adrenal crises [92, 93]. In contrast to its use in adrenal carcinoma, specific target ranges for drug concentrations in CD have not been established. Significant and frequent side effects include disturbances of the gastrointestinal tract (72 %) and the central nervous system (impaired mentation and dizziness, 45 %), as well as gynecomastia, rash, increases in liver enzymes, and hypercholesterolemia [42]. Therefore, its use should be limited to centers with special experience. Recently, a defined preparation was licensed in Europe (Lysodren^®^, Bristol-Myers Squibb).

### Aminoglutethimide

Aminoglutethimide inhibits the side-chain cleavage of cortisol biosynthesis and a variety of steroidogenic enzymes, e.g. side-chain cleavage complex, 21-hydroxylase, 17-α-hydroxylase, 11-β-hydroxylase, aromatase, 17–20 lyase, and 18-hydroxylase [42, 75]. Aminoglutethimide appears to be somehow more effective in patients with autonomous adrenal hyperfunction or ectopic ACTH production compared to patients with CD, possibly due to ACTH stimulation overriding the adrenal blockade in the latter group. As of this writing, the compound has only limited availability worldwide.

### Etomidate

The non-opioid anesthetic etomidate induces adrenocortical suppression by dose-dependent inhibition of 11-β-hydroxylase and desmolase. It is the only such compound available for intravenous administration, thus being useful in situations where rapid control of hypercortisolism is required, or oral therapy is contraindicated. An initial bolus of 0.03 mg/kg is followed by an infusion of 0.03–0.3 mg/kg/h. Significant suppression of serum cortisol levels is observed quickly (after ~5 h), with a maximum effect at ~11 h [94–96].

### LCI699

LCI 699 is a potent inhibitor of 11-β-hydroxylase and 18-hydroxylase (aldosterone synthase) [97, 98] currently under investigation in a proof-of-concept study in patients with CD. Preliminary results [99] from 10 weeks treatment in 12 patients with mild to severe CD (UFC > 1.5xULN) showed UFC normalization in 11/12 patients, and all patients achieved the study’s primary endpoint (UFC ≤ ULN or ≥50 % reduction in UFC from baseline). Median LCI699 dose associated with UFC normalization was between 5 and 10 mg bid. LCI699 was generally well tolerated, and no serious drug-related adverse events were reported. The most common adverse events were fatigue (7/12), nausea (5/12) and headache (3/12). Four patients experienced study-drug–related hypokalemia (K^+^ < 3.5 mmol/L; min 3.1 mmol/L). Based on these promising results, evaluation of LCI699 in patients with CD is ongoing.

## Combination therapy

Interaction between somatostatin analogs and dopamine receptor agonists may allow for synergistic suppression of ACTH. Feelders et al. [11] studied 17 patients with pasireotide alone or with cabergoline and ketoconazole in CD (mean age, 45.7 years; 13 women) in an 80-day trial with normalization of urinary free cortisol (UFC) levels as the main outcome measure. Pasireotide monotherapy induced sustained normalization of UFC levels in five patients, and addition of cabergoline succeeded in normalizing an additional four patients. Despite a ≥ 50 % decrease in UFC, 8/17 patients (47 %) still had elevated UFC levels after two months on the combined treatment. The addition of low-dose ketoconazole increased the number of patients with a complete response to 88 % after a further two months.

Another study reported on the effectiveness of cabergoline (at doses of up 3 mg/week) combined with relatively low doses of ketoconazole (up to 400 mg/day) in patients with CD who were unsuccessfully treated by transsphenoidal surgery [100]. Whereas treatment with cabergoline alone for six months allowed normalization of UFC in 3/12 patients, the addition of ketoconazole to the nine patients with insufficient response normalized UFC in an additional six patients.

Combination therapy with mitotane (3–5 g/24 h), metyrapone (3–4.5 g/24 h) and ketoconazole (400–1,200 mg/24 h) as an alternative to urgent adrenalectomy has been recently reported in 11 patients with severe CD [12]. In all patients, high doses of these drugs were simultaneously started, and UFC decreased within 24–48 h. After this treatment, five patients were able to undergo surgery directed at ACTH excess. Side effects were tolerable and, interestingly, no greater than would be expected for each medication alone.

## Conclusions

Cushing’s disease is a debilitating condition, with increased morbidity and mortality if biochemical remission/cure not achieved. Transsphenoidal surgery is the first-line treatment of choice, but does not result in a cure for many patients. Medical therapy represents a potentially useful addition to the range of available treatment options for many patients with CD for whom surgery has failed or is not possible. While adrenal steroidogenesis inhibitors are clearly effective, there have been few controlled trials, and little experience with their long-term use. Clinically important side effects have also limited the therapeutic potential of available therapies, and there is a possibility of publication bias for this class of therapies. Further, drugs of this type do not target the underlying pituitary tumor, and escape of ACTH secretion may require dose adjustments to maintain efficacy. Pituitary-targeted therapies may provide both an anti-secretory and an anti-proliferative treatment. Dopamine agonists have demonstrated some efficacy in small proof-of concept studies. A new somatostatin receptor ligand, pasireotide, has shown clear biochemical and clinical response. Similarly, treatment with the glucocorticoid receptor antagonist, mifepristone demonstrated clinical improvement. Our present knowledge of combination therapy is limited to case reports and a few small proof-of-concept studies; current data indicate, however, that a multimodal pharmacologic treatment approach may offer additive or synergistic clinical benefit with acceptable tolerability. Medical therapy for CD poses unique challenges and, unlike the biochemical control and tumor shrinkage achieved in patients with prolactinomas and acromegaly, comparable results have not yet been achieved in patients with CD. However, research is progressing rapidly, and we are getting closer to a more ideal treatment for this implacable disease.
